# Deciphering Microbial Adaptation in the Rhizosphere: Insights into Niche Preference, Functional Profiles, and Cross-Kingdom Co-occurrences

**DOI:** 10.1007/s00248-024-02390-3

**Published:** 2024-05-21

**Authors:** Yansu Wang, Quan Zou

**Affiliations:** Institute of Fundamental and Frontier Sciences, University of Electronic Science and Technology of China, Chengdu, 610054 China

**Keywords:** Rhizosphere microbial community, Niche differentiation, Functional adaptation, Co-occurrence network

## Abstract

Rhizosphere microbial communities are to be as critical factors for plant growth and vitality, and their adaptive differentiation strategies have received increasing amounts of attention but are poorly understood. In this study, we obtained bacterial and fungal amplicon sequences from the rhizosphere and bulk soils of various ecosystems to investigate the potential mechanisms of microbial adaptation to the rhizosphere environment. Our focus encompasses three aspects: niche preference, functional profiles, and cross-kingdom co-occurrence patterns. Our findings revealed a correlation between niche similarity and nucleotide distance, suggesting that niche adaptation explains nucleotide variation among some closely related amplicon sequence variants (ASVs). Furthermore, biological macromolecule metabolism and communication among abundant bacteria increase in the rhizosphere conditions, suggesting that bacterial function is trait-mediated in terms of fitness in new habitats. Additionally, our analysis of cross-kingdom networks revealed that fungi act as intermediaries that facilitate connections between bacteria, indicating that microbes can modify their cooperative relationships to adapt. Overall, the evidence for rhizosphere microbial community adaptation, via differences in gene and functional and co-occurrence patterns, elucidates the adaptive benefits of genetic and functional flexibility of the rhizosphere microbiota through niche shifts.

**Supplementary Information:**

The online version contains supplementary material available at 10.1007/s00248-024-02390-3.

## Introduction

Plant and rhizosphere microbiota form a complex microecosystem. Plant exudates attract a diverse range of microorganisms from the surrounding soil into the rhizosphere. The continual radial gradients spanning from the plant roots to the soil establish the environmental niche axes, which clearly influence the coexistence and migration of local populations [1–3]. An outstanding question that remains for researchers is how populations evolve in ever-changing environments. The adaptation of plants and animals through natural selection has been extensively studied, with the genome playing a crucial role in the evolutionary process. Gene flow, for instance, can override selection and lead to the conservation of species’ ranges over time [4]. However, studies of the genomic and functional adaptability of the rhizosphere microbiota are limited so far. Some specialized microbial taxa are enriched in the rhizosphere and exhibit changes in their ecological niches. An important ecological question that remains unanswered is what shapes community compositions in the rhizosphere region? While previous studies have focused mainly on the influence of external environmental factors (e.g., root exudates and soil properties) on rhizosphere microbes [5–8], they often overlook the variations caused by internal factors. Our aim was to determine the assembly strategies of rhizosphere microorganisms based on the ecological fitness theory that takes into account how species are impacted by environmental changes, which may be linked to genetics, functional traits, and cooperation or competition abilities [9]. In our study, we considered three aspects of adaptative variations: genomic structure, functional profiles, and co-occurrence networks.

We hypothesize that microbes in the bulk soil reservoir harbor the “internal power” to adapt or co-adapt to the rhizosphere. More specifically, genetic variation and covariation are important for survival when populations are exposed to environmental changes [10]. In bacteria, the exchange of genetic material and molecules can occur even between distantly related taxa, suggesting that ecological traits may be strain-specific and that closely related bacteria may occupy different ecological niches [11]. Furthermore, different genetic lineages of bacteria in high taxonomic levels share general life strategies or traits, show “ecological coherence,” and respond similarly to variations in environmental conditions [12]. Previous analyses have explored niche differentiation among closely related marine bacteria based on amplicon sequence variants (ASVs) at the genus level or at higher taxonomic levels [13, 14], suggesting that 16S rRNA gene hypervariable regions can be used to define ecological distributions at the taxonomic level. Hence, in this study, the potential distribution and fitness strategies of microbes in complex rhizosphere environments were investigated by analyzing hypervariable regions.

Functional adaptation typically occurs over long evolutionary times, but a transplant experiment demonstrated rapid evolution of a soil bacterium [15]. The stability, turnover, and diversity of microbial communities are determined by environmental perturbations and substantial differences in gene content [16]. Similarly, the ecological occupation of microbes in plant rhizosphere soils is influenced by environmental niche axes and is correlated with the physiological properties of species. These organisms constantly face the challenge of distinguishing environmental fluctuations and learning from stochastic feedback in an unstable habitat. The ability to do so can provide advantageous traits, such as high rates of reproduction, resource consumption, and ATP production, for certain rhizosphere microbes. These traits play a crucial role in determining the survival, growth, and reproduction of organisms in the rhizosphere [17].

There is intense competition among microorganisms within the community, where certain microbes can outcompete others by rapidly consuming nutrients or producing antibiotic metabolites [18]. However, not all interactions between microorganisms are competitive. Microbial communities often coevolve as they adapt to specific nutritional conditions [19]. Bacteria can communicate thanks to the diverse array of secreted biological signals. In the rhizosphere, bacteria are known to produce *N*-acyl-homoserine lactones (AHLs), the most studied quorum sensing signals, which act as signaling molecules for bacterial communication to coordinate their activities [20]. At the cross-kingdom coexistence level, the presence of various fungi mediates links between different organisms and ecosystems, with the potential to affect the macroecology and evolution of those organisms [21]. Bacteria face limitations in long-distance distribution and information exchange in soils. However, fungal hyphal networks provide a new and widespread mechanism for bacterial communication and pairwise interactions [22–24]. In this study, we investigated the microbial functional signatures that support microbial survival in rhizosphere soils and explored the mediating role of fungi in the fungal–bacterial ecological co-occurrence network.

Given that the assembly of rhizosphere microbes remains largely unknown, meta-analyses with aggregate data were utilized in this study to investigate the level of niche differentiation at the ASV level and the response of microbial function profiles to environmental heterogeneity. Important challenges addressed in this study include identifying how similar the niche is among closely related ASVs within different genera, gaining insight into how species adapt functionally to environmental niche axes and determining whether the co-occurrence network between fungi and bacteria facilitates the niche adaptation.

## Methods

### Data Collection

The soil microbiome dataset from 114 publications included 4150 bacterial and 1652 fungal soil samples using the keywords such as “microbiome,” “rhizosphere microbiome,” “soil microbiome,” “soil microbial/fungal/bacterial/diversity,” and “rhizosphere microbial community structure” in Google Scholar and PubMed. The community structure was monitored using 16S and 18S rRNA and ITS gene amplicon sequencing, with most of the sequencing data being obtained via Illumina platforms. The specific regions of focus for 16S rRNA amplicon sequencing were V3-V4/V4/V4-V5/V5-V6-V7, using primer pairs such as 341F/805R, 515/806R, 27F/805R, or 799F/1193R. The fungal ITS and 18S rRNA gene regions were amplified using the primers ITS1F/ITS2R, gITS7/ITS4, or 0817F/1196R. Details on publication-related information, NCBI Sequence Read Archive (SRA) accession numbers, amplification locations, and primers are available in Table S1-S3.

### Sequence Processing and Analysis

The amplicon sequence analysis was conducted using QIIME 2 [25]. Sequences were first subjected to quality control using the q-score filter (–p-min-quality = 4), followed by denoising and removal of chimeras using the DADA2 pipeline at default settings (qiime dada2 denoise-paired) [26]. QIIME2 was utilized to set the sampling depth at 7000 for bacteria and 4000 for fungi. After data quality control and resampling, 3419 bacterial (including 1804 bulk samples and 1615 rhizosphere samples) and 1651 fungal (including 726 bulk samples and 925 rhizosphere samples) samples were retained. The filtered sequences were identified as amplicon sequence variants (ASVs), and an ASV table was generated for each sample (feature-table summarize and feature-table tabulate-seqs). A phylogenetic tree was generated for diversity analyses (qiime alignment mafft, qiime alignment mask, and qiime phylogeny fasttree). To reduce potentially spurious and transient ASVs, singleton ASVs and those that were present in only one sample were excluded. Taxonomic assignment of the ASVs was performed against a full-length reference database, accounting for the differences in amplification regions. The bacterial/fungal full-length reference database was established by combining the RDP, SILVA, GreenGenes, and UNITE database [27–30]. RDP provides quality control, comparison, and annotation of bacterial and archaeal 16S rRNA gene and fungal 28S rRNA gene sequences. The SILVA database provides comprehensive, high quality, and regularly updated data for sequences of RNAs of bacteria, archaea, and eukaryotes (16S/18S SSU and 23S/28S LSU). GreenGenes is the comprehensive 16S rRNA gene reference database. The UNITE database is currently the most comprehensive fungal ITS collation database. Once we obtained the sequences for each database, we created a search database using the “makeblastdb” command in BLAST + v2.13.0.

### Determination of Differential and Relative Abundances of Species

Taxa importance was determined using a random forest algorithm for feature selection in the bulk and rhizosphere microbial communities. The metadata were divided into a training set (70%) and a test set (30%) for model building and evaluation, respectively. The mean decrease in prediction accuracy was used to identify genera that could serve as biomarkers to distinguish between the bulk and rhizosphere soils. All the analyses were conducted using the “RandomForest” package in R statistical software [31]. Additionally, the “edgeR” package in R was utilized to identify taxa that exhibited significant changes in rhizosphere soil samples compared to bulk soil samples [32].

The abundances of taxa were quantified according to two criteria: the relative abundances and occurrence of microbial taxa across samples. Occurrence indicates the species distribution range. We selected and retained the highly abundant ASVs with a mean relative abundance greater than 0.01% and occurrence frequency greater than 15% for bacteria or 10% for fungi across all samples. This selection was made by accounting for the diversity of sample sources and the highest frequency of occurrence observed (31.94% for bacteria and 25.92% for fungi).

### Niche Preference Analysis of Bacterial Communities

To examine the niche preference of a given genus covary, we used the Rho value between pairs of ASVs to represent the niche similarity parameter as described in a previous study [13]. The Rho value is used to analyze the associations where pairs of values behave proportionally across observations from relative or compositional data. Correlation is a commonly used statistical measure of pairwise association but should not be used on data that carry only relative information; the correlation of relative abundances can lead to conclusions opposite to those drawn from absolute abundances using correlation. As the strength of proportionality between two variables can uncover meaningful and interpretable associations in relative data, the propr v4.2 package was used to calculate the Rho value to avoid the pitfalls of analyzing correlation-like measurements in compositional data [33]. Within each genus, pairwise distances between pairs of ASVs were calculated using the function “dist.dna” in the K80 model of the “ape” package [34]. To ensure a close relationship between the two ASVs, the nucleotide distance threshold was set to 0.5, as numerous genera exhibited considerable gaps in nucleotide distances **(**Fig. S1**)**. We analyzed the closely related bacterial ASVs of 14 genera that differed significantly between the bulk and rhizosphere soils. A linear model was utilized to test which genera exhibited a significant relationship between nucleotide divergence and Rho.

### Functional Profile Analysis of Bacterial Communities

Functional profiles were predicted using Tax4fun2 based on the ASVs with the Ref99NR database [35]. The functional redundancy indices (FRIs) were calculated using the proportions of species with specific functions and their phylogenetic relationships. A high FRI indicates greater functional redundancy, indicating that the function is widespread among microbial members, while a low FRI indicates that the function is present only in some closely related species or is detected in only one species. An FRI of 0 indicates that the function is absent. The absolute FRI (aFRI) is normalized using the mean phylogenetic distance of all prokaryotes in Tax4Fun2 reference data; therefore, the aFRI allows comparisons of functional redundancy in different ecosystems.

### Co-occurrence Network Construction and Keystone Species Identification

The correlation matrix between all possible pairs of ASVs was converted into an adjacency matrix. The cutoff of correlation coefficients was determined to be 0.6, with a significance threshold of *p* < 0.001, using the random matrix theory (RMT)-based method [36], which has been widely used to construct microbial co-occurrence networks [37–40]. The network was constructed and visualized using ggClusterNet [41]. The keystone taxa were defined using values of within-module connectivity (*Z*_*i*_) and among-module connectivity (*P*_*i*_), which included module hubs (*Z*_*i*_ > 0.25, *P*_*i*_ ≤ 0.62), connectors (*Z*_*i*_ ≤ 0.25, *P*_*i*_ > 0.62), and network hubs (*Z*_*i*_ > 0.25, *P*_*i*_ > 0.62). These keystone taxa are thought to have a significant effect on microbial community structure [42]. Betweenness centrality (BC) refers to the number of shortest paths that go through a given node. The betweenness centrality of node *i* is calculated as shown in Eq. 1:

1 $$\begin{array}{c}{BC}_{i}=\sum_{s\ne i\ne t}\frac{{n}_{st}^{i}}{{g}_{st}}\end{array}$$

${n}_{st}^{i}$ represents the number of paths passing through node *i*, while ${g}_{st}$ indicates the number of shortest paths between nodes *s* and *t*.

Closeness centrality (CC) is used to measure the importance of a node and refers to how easy it is for the node to reach other nodes; the relevant formulas are shown in Eqs. 2 and 3:

2 $$\begin{array}{c}d_i=\frac1{N-1}\sum_{j=1}^Nd_{ij}\end{array}$$

3 $$\begin{array}{c}{CC}_{i}=\frac{1}{{d}_{i}}\end{array}$$

${d}_{i}$ is the average shortest path distance between node *i* and all other reachable nodes. Closeness centrality is the reciprocal of ${d}_{i}$.

### Statistical Analyses

Alpha and beta diversity indices of microbial community in each sample were calculated using QIIME2 (qiime diversity core-metrics). Community similarity was assessed using nonmetric multidimensional scaling (NMDS) based on the Bray–Curtis distance (metaMDS function of the vegan package). The Wilcoxon test was used for statistical comparisons between independent nonparametric groups. The Wilcoxon test was performed using the function “wilcox.test” from the stats package. All these analyses were visualized in R using the ggplot2 package (https://cran.r project.org/web/packages/ggplot2/index.html) in R.

## Results

### Niche Differentiation of Closely Related ASVs Within Genera

**Fig. 1:**
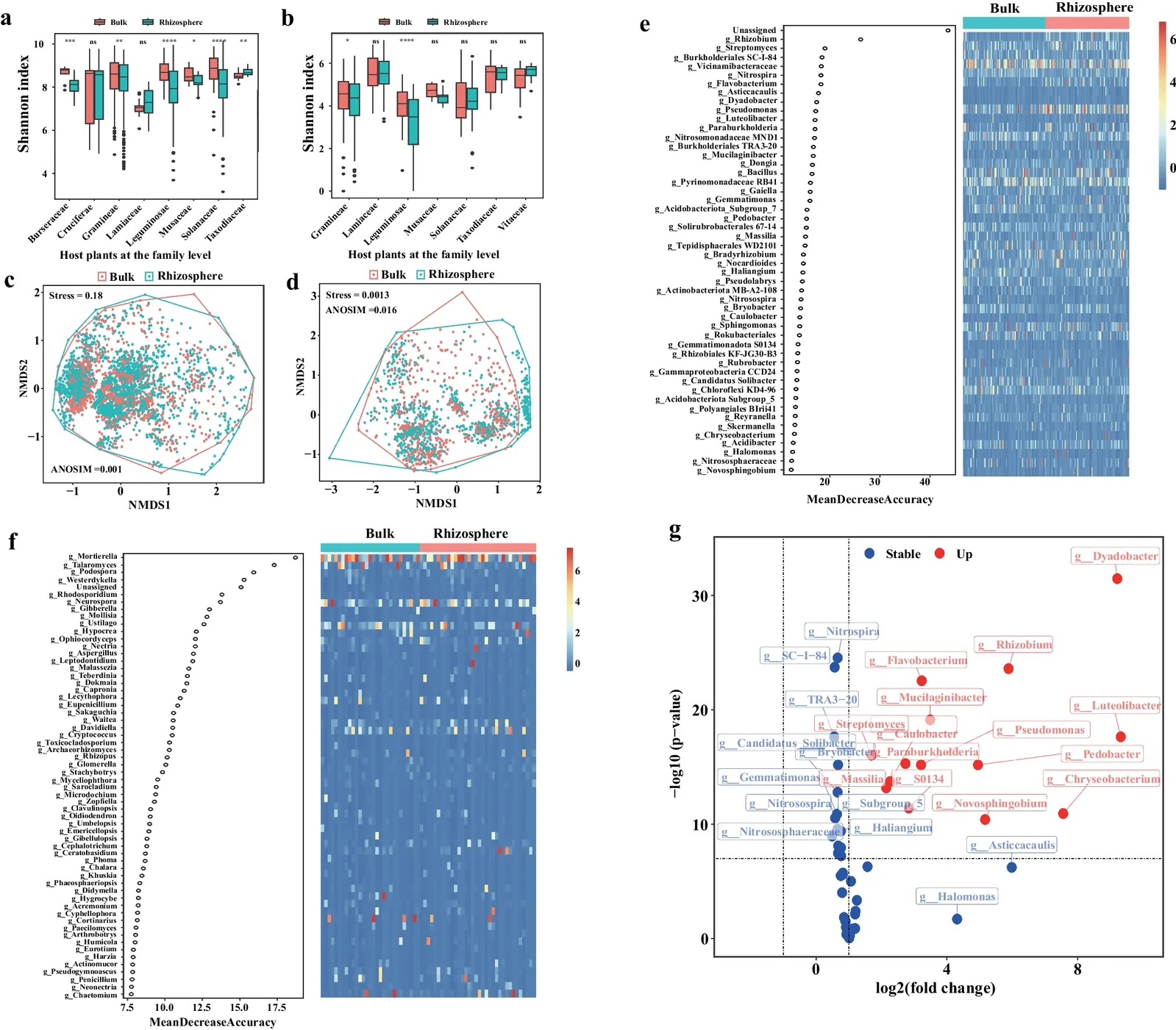
Microbial community structure in bulk and rhizosphere soils. **a**–**d** Alpha (**a**, **b**) and beta (**c**, **d**) diversity of bacteria (**a**, **c**) and fungi (**b**, **d**) in bulk and rhizosphere soils. The *x*-axis for **a** and **b** is the different host types at family level for rhizosphere soils (*P < 0.05; **P < 0.01; ***P < 0.001). The heatmap scale represents the abundance of bacterial (**e**) and fungal (**f**) indicators. The color scale on the right side of the heatmap illustrates the normalized abundance. Differential genera of bacteria are marked in red font in volcano plots (**g**)

The alpha diversity of both bacteria and fungi was higher in the bulk soil compared to the rhizosphere soil, with the exception of *Taxodiaceae* and *Vitaceae*. There are significant differences in alpha diversity of both bacteria and fungi between the bulk soil and rhizosphere soil of plants such as *Burseraceae*, *Gramineae*, *Leguminosae*, *Musaceae*, and *Solanaceae* (*p* < 0.05, Wilcoxon rank-sum test) (Fig. 1a, b). Together, the beta diversity analysis reflects the differences in the microbial community structure among different host plants **(**Table S4 and Table S5**)**, and the NMDS score plot reveals the dissimilarity of community composition between bulk and rhizosphere soils based on Bray‒Curtis dissimilarity matrices **(**Fig. 1c, d, ANOSIM = 0.001 and 0.016).

To delineate the potential driving mechanism behind the ecological niche distribution of a given taxon, we focused on biomarkers and differential taxa as research subjects. Random forest analysis was used to determine the contributions of different genera to the classification of the bulk and rhizosphere soils and to identify indicator taxa between the bulk soils and rhizosphere soils. The cross-validation curve describes the relationship between the model error and the number of ASVs **(**Fig. S2**)**. The model error clearly decreased as the number of ASVs increased, but the rate of decrease slowed and eventually became constant. Based on the cross-validation curve, the 50 most abundant bacterial ASVs and the 60 most abundant fungal ASVs were selected as indicator genera. The highest-ranking genera for bacteria and fungi were *Rhizobium* and *Mortierella*, respectively, except for unassigned taxa **(**Fig. 1e, f**)**. Log2-fold changes and *t* tests were used to identify the indicator genera with significant differences in abundance between the bulk and rhizosphere samples. While no differential fungal genera were detected **(**Fig. S3**)**, 14 differential bacterial genera were detected with higher average relative abundances in the rhizosphere than in the bulk soil. These genera were visualized using volcano plots **(**Fig. 1g**)**.

**Fig. 2:**
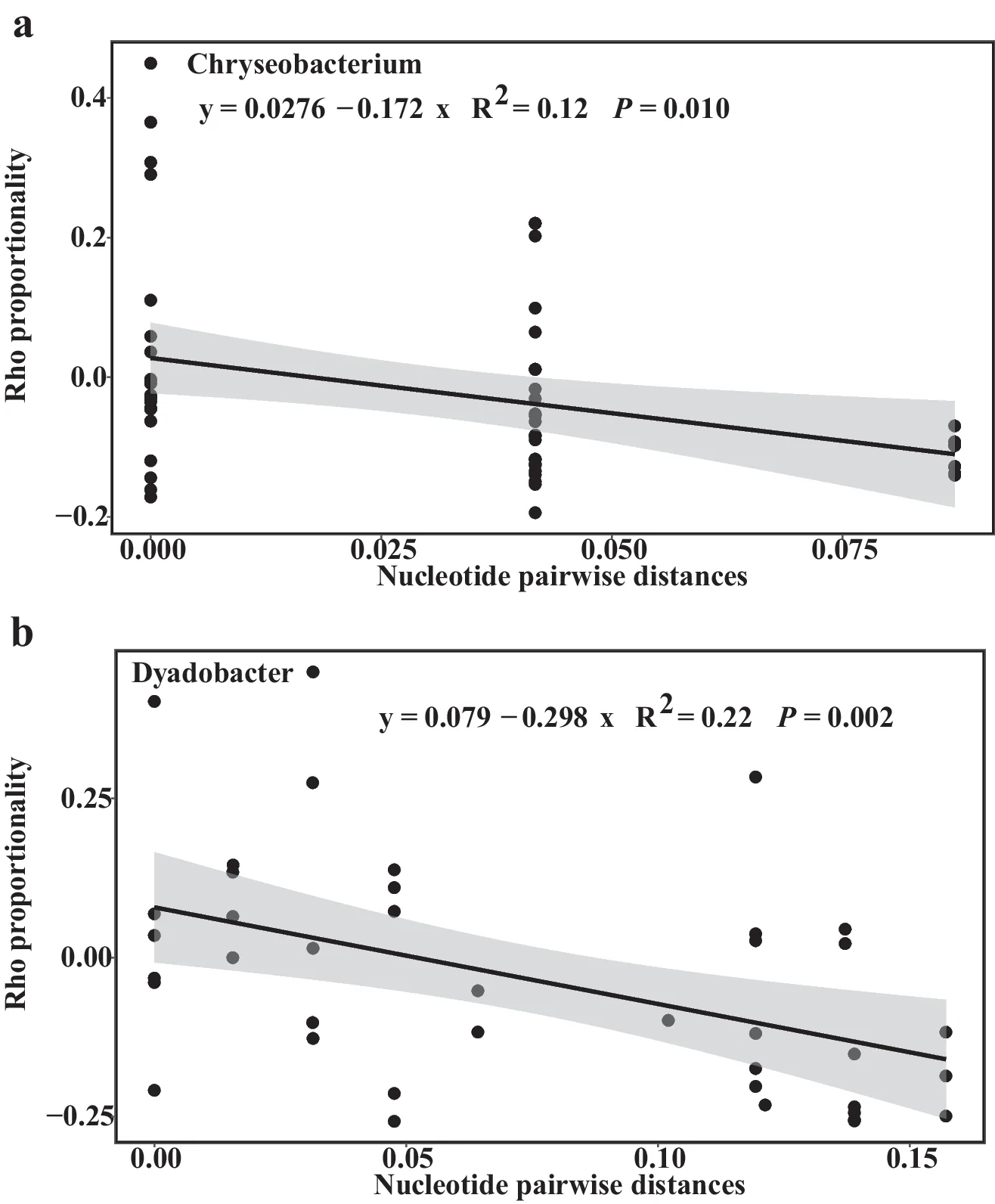
Relationship between the Rho proportionality and the nucleotide divergence. The closely related ASV is defined as a nucleotide distance less than 0.5. Gray and black lines represent the linear relationship between two variables. The *P* value is displayed for the regressions

We next defined the ecological niches of the ASVs from the 14 differentially abundant genera. The Rho values were used to measure niche overlap among closely related ASVs, and pairwise distances of DNA sequences were computed to indicate nucleotide divergence. Among the 14 bacterial genera, the Rho proportions of closely related ASVs exhibited different tendencies, suggesting that the changes are specific to different groups (Fig. 2 and Fig. S4). The genera *Chryseobacterium* and *Dyadobacter* exhibit niche differentiation as nucleotide divergence increases (Fig. 2a, b), suggesting that trait variation among ASVs could be attributed to local adaptation to environmental conditions. Although the correlation between niche differentiation and nucleotide divergence of the three species *Streptomyces*, *Pseudomonas*, and *Pedobacter* has a *P*-value of less than 0.05, the *R*^2^ value is small, making it impossible to accurately infer the relationship between niche differentiation and nucleotide divergence. Therefore, these three species are not included in the discussion (Fig. S4 j-l).

### Enhanced Metabolism of Biological Macromolecules and Cell‒Cell Communication in Abundant Bacteria in the Rhizosphere

**Fig. 3:**
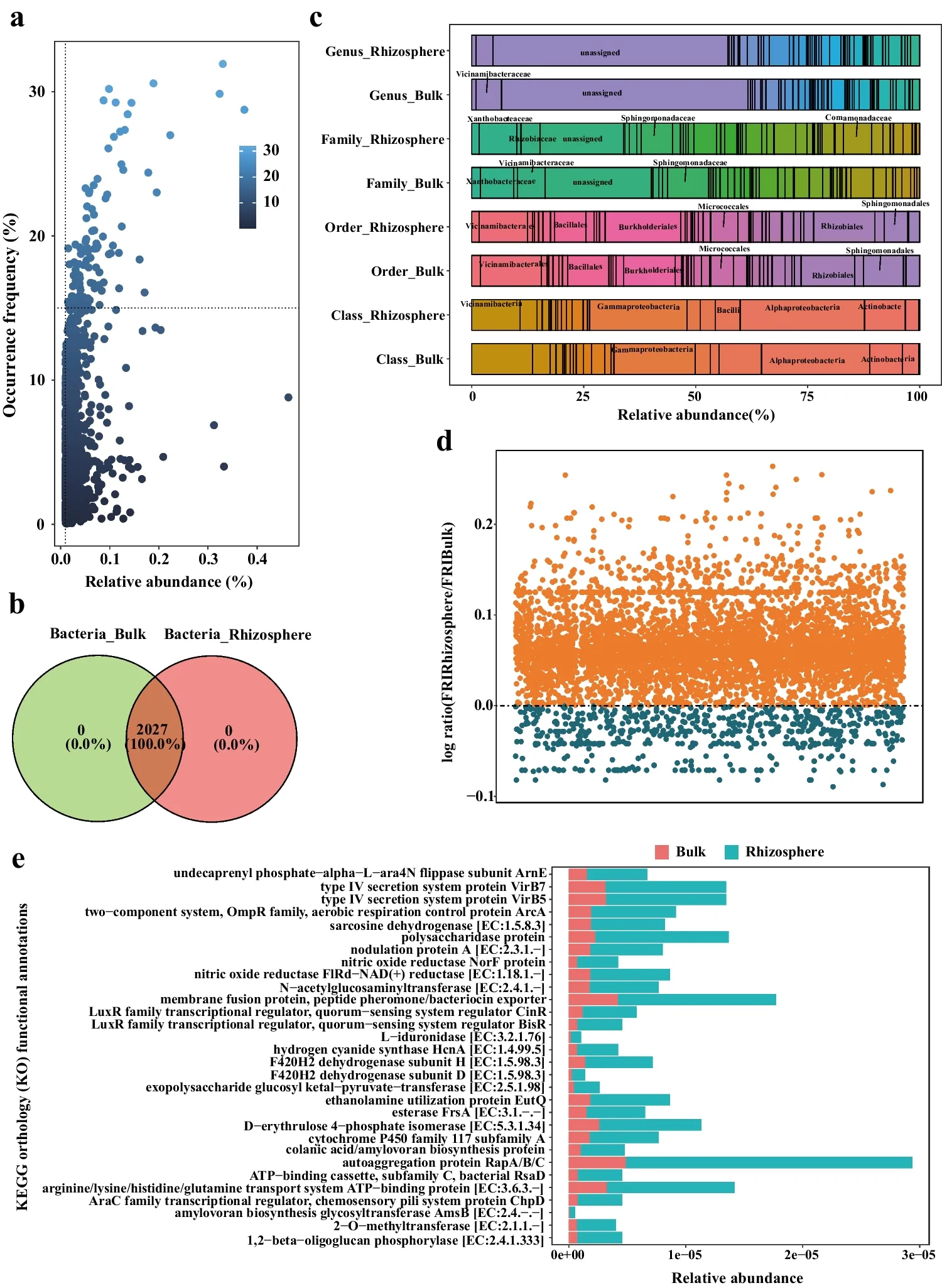
Function profiles of bacterial abundant species. The bacterial abundant ASVs are defined as the relative abundance and occurrence frequency (**a**), and the Venn diagram shows the shared ASVs in bulk and rhizosphere samples (**b**). **c** Bar chart showing the relative abundance of abundant bacterial species at various taxonomic levels in bulk and rhizosphere samples. Larger squares indicate higher taxa abundance, with specific taxa of greater abundance labeled on the plot. **d** Functional redundancy indices of bulk (blue) and rhizosphere (orange) samples. A log ratio greater than 0 indicates that a function is more redundant in rhizosphere soils. **e** Top 30 significant different functions of the abundant ASVs

The abundant taxa are defined by their occurrence frequency and relative abundance to study how these species adapt to variations in different habitats (Fig. 3a, b and Fig. S5a and c), and Tables S6 and S7 display the taxonomic information of these abundant taxa. Most of these bacterial ASVs were classified as *Alphaproteobacteria*, *Gammaproteobacteria*, or *Vicinamibacteria* at the class level (Fig. 3c). The high-abundance ASVs of fungi belonged to *Sordariomycetes*, *Mucoromycotina*, and *Dothideomycetes* (Fig. S5b). Then, the functional assignments of these abundant ASVs were performed, 6937 KOs were predicted for bacteria, and 859 KOs were predicted for fungi. The functional profiles of the abundant fungal ASVs were not significantly different between the bulk and rhizosphere soils (Fig. S5d-e); therefore, the focus was on the functional profiles of the abundant bacterial ASVs. The aFRI was applied to compare functional redundancy between bulk and rhizosphere microbes, and a higher aFRI indicates that the function is extensive in the community members. Figure 3d shows that the rhizosphere microbes had a greater number of functions with functional redundancy than did the bulk microbes, which suggested that the members of the rhizosphere soils underwent functional changes in response to the microenvironment or substrate renewal in the habitat. A total of 334 bacterial KOs were significantly enriched in the rhizosphere samples (Fig. S6), and the differentially abundant KOs were involved in pathways related to carbohydrate metabolism, amino acid transport, and energy conversion (Fig. 3e). Notably, the autoaggregation protein Rap A/B/C was the most distinguishing function, as it plays a crucial role in bacterial autoaggregation, which is linked to surface colonization and biofilm formation.

### Cross-Kingdom Co-occurrence Network Fluctuations with Habitat Changes

**Fig. 4:**
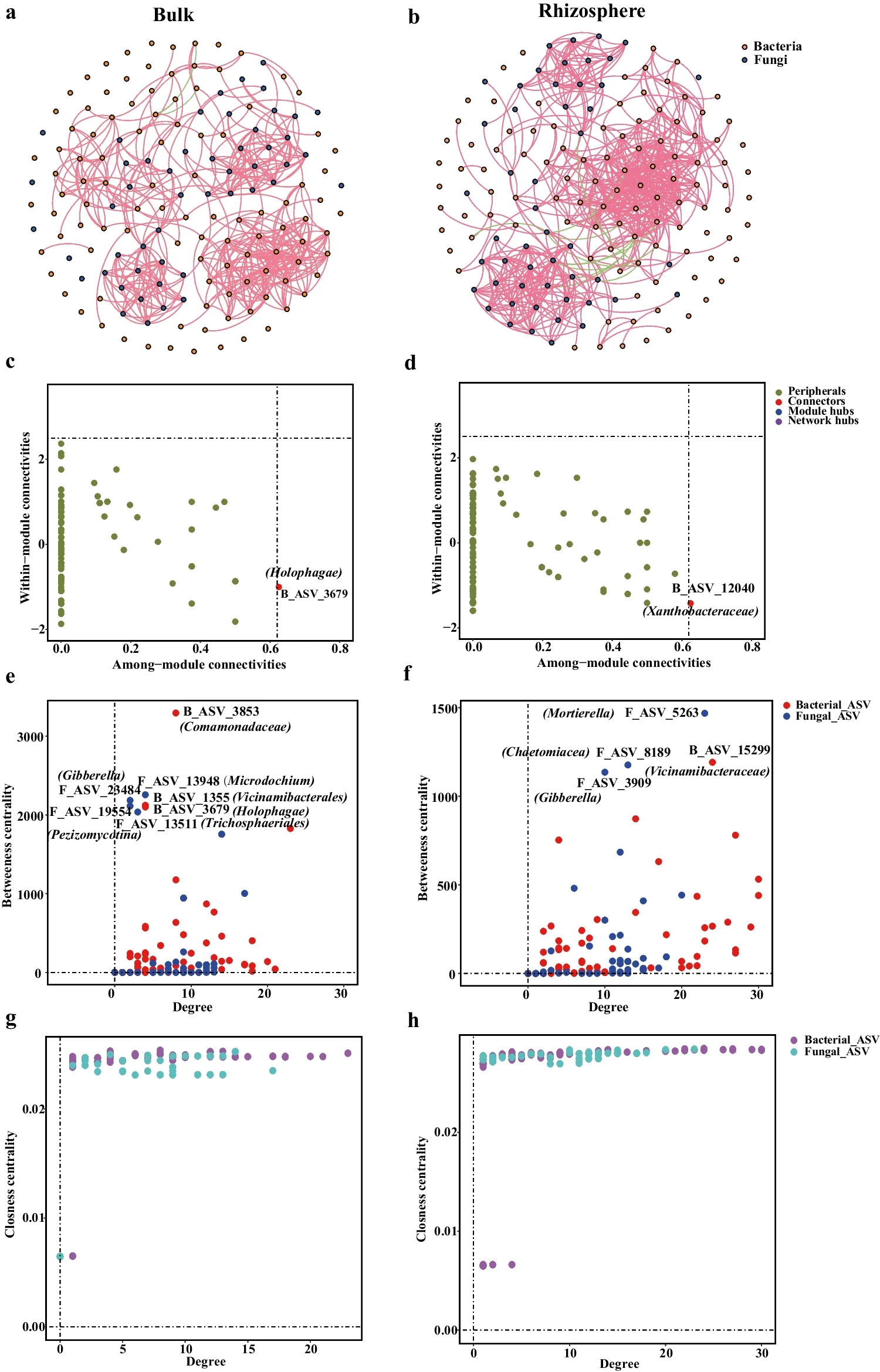
Cross-kingdom co-occurrence networks of abundant microbes for bulk (**a**, **c**, **e**, **g**) and rhizosphere (**b**, **d**, **f**, **h**) samples. The Z–P plot shows node categories according to within‐module connectivity (*Z*_*i*_) and among‐module connectivity (*P*_*i*_) (**c**, **d**). The scatter plots represent the distribution of node betweenness centrality (**e**, **f**) and closeness centrality (**g**, **h**)

To explore the co-occurrence patterns of abundant microbes in the bulk and rhizosphere soils, we used network analysis and constructed a network with abundant microbes (Fig. 4a, b). The rhizosphere and bulk networks contained one connector each, belonging to the *Acidobacteriota* and *Proteobacteria* phyla, respectively. No module hubs or network hubs were identified in either network (Fig. 4c, d). Bacterial ASV_12040 (*Xanthobacteraceae*) shifted from a peripheral role to a connector role, linking modules in the rhizosphere network (Fig. 4d). To investigate the relationship between bacteria and fungi, two centrality measures, namely, betweenness centrality and closeness centrality, were determined. Betweenness centrality refers to the number of times a node acts as the shortest bridge between two other nodes. A node with higher betweenness centrality is regarded as a social intermediary that maintains the connections between two nodes. Figure 4 e and f and Table S8 demonstrate that fungal ASVs play a more significant role than bacterial ASVs as intermediaries in both bulk and rhizosphere soils, with betweenness centrality thresholds of 2000 and 1000, respectively, while there was no significant difference between bacteria and fungi with higher closeness centrality, which is a measure of key node centrality in the network (Fig. 4g, h). Additionally, the average degree measures the network complexity, and the average path length quantifies the level of integration in the network. Figure S7 shows that the rhizosphere soils exhibited enhanced cross-kingdom complexity and connectivity, with a lower average node degree in the bulk soil (5.87) than in the rhizosphere soil samples (8.23) and a greater average path length in the bulk soil (6.12) than in the rhizosphere soil samples (3.54). Thus, we believe that cross-kingdom co-occurrence networks may adaptively fluctuate in response to changes in the environment and that fungal communities can influence bacterial co-occurrence relationships.

## Discussion

The evolution of niche breadth plays a crucial role in species adaptation and speciation, allowing them to shift their distributional ranges or adapt to environmental changes [43]. Understanding whether species switch their niches between bulk and rhizosphere soils and how niche breadth divergently evolves among related taxa is an important topic in the study of adaptive plasticity of rhizosphere microbes. As co-occurrence and covariance can characterize niche similarity, we investigated niche overlap among closely related taxa using the Rho measurement described by Auladell, et al. [13]. We found a clear trend between niche similarity and nucleotide divergence for *Chryseobacterium* and *Dyadobacter*, in which there was significantly lower niche similarity along with nucleotide divergence, as shown by a pattern similar to that of environmental filtering [40]. These findings highlight the diversity of ecological trends, wherein some closely related ASVs share similar ecological traits in response to habitat changes [44]. In fact, not all genera exhibit a remarkable relationship between Rho and nucleotide divergence. A possible explanation is that genetic variations in the 16S rRNA region could not reflect the genomic differences among these genera. Increases in sequencing length, particularly metagenome sequencing, could provide the opportunity to obtain a clearer picture of microbial niche adaptation mechanisms.

There is more evidence indicating that genetic variation contributes to the variation in functional traits at fine genetic scales [45, 46]. Therefore, further analysis of functional traits was performed to explore microbial fitness in the rhizosphere, which is believed to be closely associated with organism features rather than microbial composition. Our study revealed that functions related to carbohydrate and amino acid metabolism in the abundant microorganisms were enriched under rhizosphere conditions, as plant roots provide ample carbon and nitrogen sources to the rhizosphere [17, 47–50]. The abundant microorganisms are typically habitat social generalists whose genomes encode a wide range of functions, reflecting the flexibility in resource usage that allows them to survive in a variety of niches [49, 51]. The ability to metabolize multiple carbon sources, such as organic carbon and carbon dioxide, and electron acceptors, including hydrogen, formate, and conductive materials, either simultaneously or alternately, in many microorganisms aids them in maintaining metabolic diversity during environmental disturbance and contributes to the resistance and resilience of a community [52–54].

In addition, our results revealed that certain functions, such as those involved in the regulation of the quorum sensing system, secretion system proteins, membrane fusion proteins, and autoaggregation proteins, are increased in the rhizosphere. This suggests that bacterial communication through quorum sensing is enhanced in the rhizosphere. Quorum sensing regulates group-coordinated behavior via small molecules, and this process controls various phenomena, such as virulence factor secretion [51], biofilm formation [55], and DNA transfer [56]. Our results imply that the rhizosphere environment promotes the sophisticated cooperative behaviors of bacteria, which allows for population density–dependent advantageous lifestyles and increases bacterial survival. However, the interconnection and modulation of microbial quorum sensing signals, which are influenced by environmental factors (especially host plants) and spatiotemporal constraints, require further elucidation.

In addition to abiotic parameters, biotic interactions such as mutualism and competition also play crucial roles in determining the survival of species in a particular environment. Therefore, it is imperative to observe and understand the rapid adaptive responses of species to these interactions. Considering the involvement of fungi in resource and energy cycles, which promote bacterial nutrient uptake and growth [57], we constructed a cross-kingdom co-occurrence network to determine the role of fungi in bacterial fitness. Overall, the abundant microorganisms in the rhizosphere form a more complex network with densely connected nodes, where bacteria (the subgroups *Holophagae* and *Xanthobacteraceae*) act as connectors that link modules, substantially impacting the stability of the microbial network. *Xanthobacteraceae* belongs to the order *Rhizobiales* and can utilize various C substrates [58, 59], which supports their key ecological roles in maintaining network structure. Moreover, our further work revealed that certain fungi, such as *Microdochium*, *Mortierella*, and *Gibberella*, display greater betweenness centrality in both the bulk soil and rhizosphere, indicating that they are responsible for a greater number of shortest paths. These findings are consistent with the notion that mycelium-driven bacterial dispersal [60] occurs because these fungi are bridge nodes and serve as intermediary agents that contribute to maintaining bacterial coexistence patterns.

## Conclusions

**Fig. 5:**
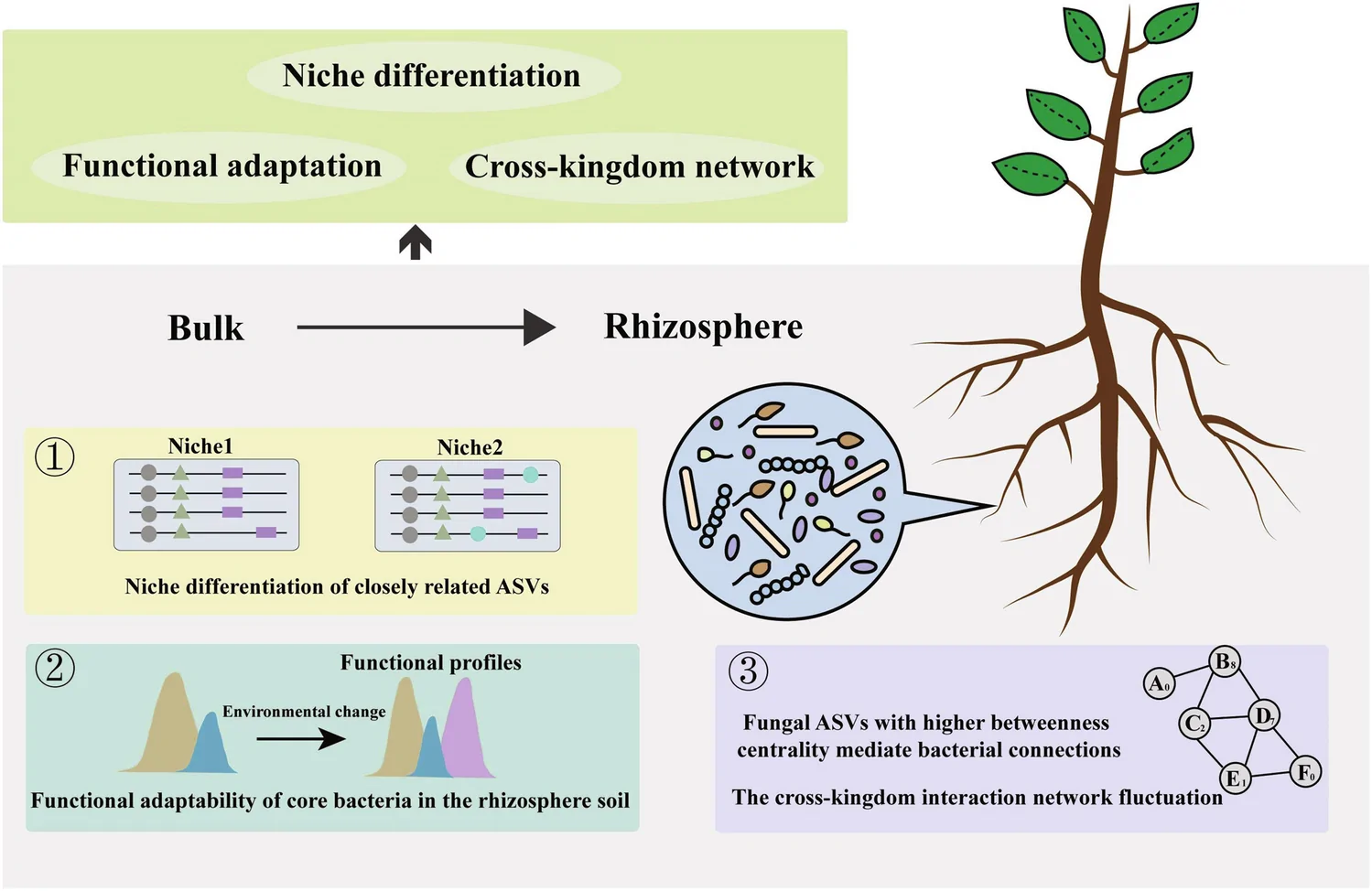
Overview of mechanisms of microbial adaptation in the rhizosphere soil. The conceptual paradigm showing the linkage, the niche differentiation of closely related ASVs, the functional profile changes, and the cross-kingdom co-occurrence pattern fluctuation along with habitat changes

In conclusion, our study provides insight into how rhizosphere microorganisms adapt to plant rhizosphere soil through three aspects of adaptive biology (Fig. 5). Firstly, we observed a clear pattern revealing a notable correlation between niche similarity and nucleotide divergence among closely related ASVs of *Chryseobacterium* and *Dyadobacter*. This sheds light on the nucleotide divergence that drives niche differentiation among closely related organisms. Secondly, in addition to the discovery of the common phenomenon of enrichment of carbohydrate and amino acid metabolism in the rhizosphere, it is noteworthy that bacterial quorum sensing–related protein genes are significantly enriched in rhizosphere soil, implying the enhanced microbial communication in the rhizosphere. Thirdly, the establishment of a cross-kingdom co-occurrence network underscores the significance of cross-species collaborative interactions. The results align with the notion that mycelium-driven bacterial dispersal occurs, with fungi serving as bridging nodes that support the maintenance of bacterial coexistence patterns. To unravel the adaptive and evolutionary mechanisms of microbial populations in response to environmental fluctuations, further analysis is warranted, such as assigning sequence variants to metagenome-assembled genomes (MAGs) or meta-transcriptomes.

## Supplementary Information

Below is the link to the electronic supplementary material.

Supplementary file1 (XLSX 923 KB)

Supplementary file2 (DOCX 895 KB)

## Data Availability

All raw data is archived under NCBI BioProject and their accession numbers are available in Supplemental Table S1. All the codes used are available in the following repository: https://github.com/Wangys-prog/microbial_diversity.

## References

[1] Chialva M, Lanfranco L, Bonfante P (2022) The plant microbiota: composition, functions, and engineering. Curr Opin Biotechnol 73:135–142 34392234 10.1016/j.copbio.2021.07.003

[2] Gu Y, Wang X, Yang T, Friman V-P, Geisen S, Wei Z, Xu Y, Jousset A, Shen Q (2020) Chemical structure predicts the effect of plant-derived low-molecular weight compounds on soil microbiome structure and pathogen suppression. Funct Ecol 34:2158–2169. 10.1111/1365-2435.13624

[3] Rolfe SA, Griffiths J, Ton J (2019) Crying out for help with root exudates: adaptive mechanisms by which stressed plants assemble health-promoting soil microbiomes. Curr Opin Microbiol 49:73–82. 10.1016/j.mib.2019.10.003 31731229 10.1016/j.mib.2019.10.003

[4] Lenormand T (2002) Gene flow and the limits to natural selection. Trends Ecol Evol 17:183–189

[5] Dastogeer KM, Tumpa FH, Sultana A, Akter MA, Chakraborty A (2020) Plant microbiome–an account of the factors that shape community composition and diversity. Current Plant Biology 23:100161

[6] Bourceret A, Guan R, Dorau K, Mansfeldt T, Omidbakhshfard A, Medeiros DB, Fernie AR, Hofmann J, Sonnewald U, Mayer J, Gerlach N, Bucher M, Garrido-Oter R, Spaepen S, Schulze-Lefert P (2022) Maize field study reveals covaried microbiota and metabolic changes in roots over plant growth. mBio 13:e02584-e2521. 10.1128/mbio.02584-21 10.1128/mbio.02584-21 PMC9040757 35258335

[7] Zhu YL, Huang YJ, Nuerhamanti N, Bai XY, Wang HN, Zhu XY, Zhang W (2023) The composition and diversity of the rhizosphere bacterial community of ammodendron bifolium growing in the Takeermohuer Desert are different from those in the nonrhizosphere. Microb Ecol 87:2. 10.1007/s00248-023-02320-9 38008827 10.1007/s00248-023-02320-9 PMC13498302

[8] Gu Y, Liang W, Li Z, Liu S, Liang S, Lei P, Wang R, Gao N, Li S, Xu Z, Xu H (2023) The biocontrol agent *Bacillus velezensis* T-5 changes the soil bacterial community composition by affecting the tomato root exudate profile. Plant Soil 490:669–680. 10.1007/s11104-023-06114-3

[9] Peacock KA (2011) The three faces of ecological fitness. Studies in History and Philosophy of Science Part C: Studies in History and Philosophy of Biological and Biomedical Sciences 42:99–105. 10.1016/j.shpsc.2010.11.011 10.1016/j.shpsc.2010.11.011 21300321

[10] Sæther B-E, Engen S (2015) The concept of fitness in fluctuating environments. Trends Ecol Evol 30:273–281 10.1016/j.tree.2015.03.007 10.1016/j.tree.2015.03.007 25843273

[11] Arnold BJ, Huang I-T, Hanage WP (2022) Horizontal gene transfer and adaptive evolution in bacteria. Nat Rev Microbiol 20:206–218 34773098 10.1038/s41579-021-00650-4

[12] Philippot L, Andersson SG, Battin TJ, Prosser JI, Schimel JP, Whitman WB, Hallin SJNRM (2010) The ecological coherence of high bacterial taxonomic ranks. Nat Rev Microbiol 8:523–529. 10.1038/nrmicro2367 10.1038/nrmicro2367 20531276

[13] Auladell A, Barberán A, Logares R, Garcés E, Gasol JM, Ferrera I (2022) Seasonal niche differentiation among closely related marine bacteria. ISME J 16:178–189 34285363 10.1038/s41396-021-01053-2 PMC8692485

[14] Auladell A, Sánchez P, Sánchez O, Gasol JM, Ferrera I (2019) Long-term seasonal and interannual variability of marine aerobic anoxygenic photoheterotrophic bacteria. ISME J 13:1975–1987 30914777 10.1038/s41396-019-0401-4 PMC6776013

[15] Chase AB, Weihe C, Martiny JB (2021) Adaptive differentiation and rapid evolution of a soil bacterium along a climate gradient. Proc Natl Acad Sci 118:e2101254118 33906949 10.1073/pnas.2101254118 PMC8106337

[16] Viver T, Conrad RE, Orellana LH, Urdiain M, González-Pastor JE, Hatt JK, Amann R, Antón J, Konstantinidis KT, Rosselló-Móra R (2021) Distinct ecotypes within a natural haloarchaeal population enable adaptation to changing environmental conditions without causing population sweeps. ISME J 15:1178–1191 33342997 10.1038/s41396-020-00842-5 PMC8182817

[17] Wang W, Jia T, Qi T, Li S, Degen AA, Han J, Bai Y, Zhang T, Qi S, Huang M (2022) Root exudates enhanced rhizobacteria complexity and microbial carbon metabolism of toxic plants. Iscience 25:105243 36274956 10.1016/j.isci.2022.105243 PMC9579507

[18] Babalola OO, Emmanuel OC, Adeleke BS, Odelade KA, Nwachukwu BC, Ayiti OE, Adegboyega TT, Igiehon NO (2021) Rhizosphere microbiome cooperations: strategies for sustainable crop production. Curr Microbiol 78:1069–1085. 10.1007/s00284-021-02375-2 33611628 10.1007/s00284-021-02375-2

[19] Goyal A, Bittleston LS, Leventhal GE, Lu L, Cordero OX (2022) Interactions between strains govern the eco-evolutionary dynamics of microbial communities. Elife 11:e74987 35119363 10.7554/eLife.74987 PMC8884728

[20] Faure D, Vereecke D, Leveau JHJ (2009) Molecular communication in the rhizosphere. Plant Soil 321:279–303. 10.1007/s11104-008-9839-2

[21] Bahram M, Netherway T (2022) Fungi as mediators linking organisms and ecosystems. FEMS Microbiol Rev 46:fuab058 34919672 10.1093/femsre/fuab058 PMC8892540

[22] Jansa J, Hodge A (2021) Swimming, gliding, or hyphal riding? On microbial migration along the arbuscular mycorrhizal hyphal highway and functional consequences thereof. New Phytol 230:14–16 33600606 10.1111/nph.17244

[23] Warmink J, Nazir R, Corten B, Van Elsas J (2011) Hitchhikers on the fungal highway: the helper effect for bacterial migration via fungal hyphae. Soil Biol Biochem 43:760–765

[24] Zhang Y, Kastman EK, Guasto JS, Wolfe BE (2018) Fungal networks shape dynamics of bacterial dispersal and community assembly in cheese rind microbiomes. Nat Commun 9:336 29362365 10.1038/s41467-017-02522-z PMC5780524

[25] Bolyen E, Rideout JR, Dillon MR, Bokulich NA, Abnet CC, Al-Ghalith GA, Alexander H, Alm EJ, Arumugam M, Asnicar F (2019) Reproducible, interactive, scalable and extensible microbiome data science using QIIME 2. Nat Biotechnol 37:852–857 31341288 10.1038/s41587-019-0209-9 PMC7015180

[26] Callahan BJ, McMurdie PJ, Rosen MJ, Han AW, Johnson AJA, Holmes SP (2016) DADA2: High-resolution sample inference from Illumina amplicon data. Nat Methods 13:581–583 27214047 10.1038/nmeth.3869 PMC4927377

[27] Maidak BL, Olsen GJ, Larsen N, Overbeek R, McCaughey MJ, Woese CR (1996) The Ribosomal Database Project (RDP). Nucleic Acids Res 24:82–85. 10.1093/nar/24.1.82 8594608 10.1093/nar/24.1.82 PMC145599

[28] Quast C, Pruesse E, Yilmaz P, Gerken J, Schweer T, Yarza P, Peplies J, Glöckner FO (2012) The SILVA ribosomal RNA gene database project: improved data processing and web-based tools. Nucleic Acids Res 41:D590–D596. 10.1093/nar/gks1219 23193283 10.1093/nar/gks1219 PMC3531112

[29] DeSantis TZ, Hugenholtz P, Larsen N, Rojas M, Brodie EL, Keller K, Huber T, Dalevi D, Hu P, Andersen GL (2006) Greengenes, a chimera-checked 16S rRNA gene database and workbench compatible with ARB. Appl Environ Microbiol 72:5069–5072. 10.1128/AEM.03006-05 16820507 10.1128/AEM.03006-05 PMC1489311

[30] Nilsson RH, Larsson K-H, Taylor AFS, Bengtsson-Palme J, Jeppesen TS, Schigel D, Kennedy P, Picard K, Glöckner FO, Tedersoo L, Saar I, Kõljalg U, Abarenkov K (2018) The UNITE database for molecular identification of fungi: handling dark taxa and parallel taxonomic classifications. Nucleic Acids Res 47:D259–D264. 10.1093/nar/gky1022 10.1093/nar/gky1022 PMC6324048 30371820

[31] Breiman L (2001) Random forests. Mach Learn 45:5–32. 10.1023/A:1010933404324

[32] Robinson MD, McCarthy DJ, Smyth GK (2010) edgeR: a Bioconductor package for differential expression analysis of digital gene expression data. Bioinformatics 26:139–140 10.1093/bioinformatics/btp616 10.1093/bioinformatics/btp616 PMC2796818 19910308

[33] Quinn TP, Richardson MF, Lovell D, Crowley TM (2017) propr: an R-package for identifying proportionally abundant features using compositional data analysis. Sci Rep 7:16252. 10.1038/s41598-017-16520-0 29176663 10.1038/s41598-017-16520-0 PMC5701231

[34] Paradis E, Schliep K (2018) ape 5.0: an environment for modern phylogenetics and evolutionary analyses in R. Bioinformatics 35:526–528. 10.1093/bioinformatics/bty633 10.1093/bioinformatics/bty633 30016406

[35] Wemheuer F, Taylor JA, Daniel R, Johnston E, Meinicke P, Thomas T, Wemheuer B (2020) Tax4Fun2: prediction of habitat-specific functional profiles and functional redundancy based on 16S rRNA gene sequences. Environ Microbiome 15:1–12 33902725 10.1186/s40793-020-00358-7 PMC8067651

[36] Luo F, Zhong J, Yang Y, Scheuermann RH, Zhou J (2006) Application of random matrix theory to biological networks. Phys Lett A 357:420–423. 10.1016/j.physleta.2006.04.076

[37] Ma B, Wang H, Dsouza M, Lou J, He Y, Dai Z, Brookes PC, Xu J, Gilbert JA (2016) Geographic patterns of co-occurrence network topological features for soil microbiota at continental scale in eastern China. ISME J 10:1891–1901. 10.1038/ismej.2015.261 26771927 10.1038/ismej.2015.261 PMC5029158

[38] Tu Q, Yan Q, Deng Y, Michaletz ST, Buzzard V, Weiser MD, Waide R, Ning D, Wu L, He Z, Zhou J (2020) Biogeographic patterns of microbial co-occurrence ecological networks in six American forests. Soil Biol Biochem 148:107897. 10.1016/j.soilbio.2020.107897

[39] Weiss S, Van Treuren W, Lozupone C, Faust K, Friedman J, Deng Y, Xia LC, Xu ZZ, Ursell L, Alm EJ, Birmingham A, Cram JA, Fuhrman JA, Raes J, Sun F, Zhou J, Knight R (2016) Correlation detection strategies in microbial data sets vary widely in sensitivity and precision. ISME J 10:1669–1681. 10.1038/ismej.2015.235 26905627 10.1038/ismej.2015.235 PMC4918442

[40] Wang Y, Li C, Tu B, Kou Y, Li X (2021) Species pool and local ecological assembly processes shape the β-diversity of diazotrophs in grassland soils. Soil Biol Biochem 160:108338. 10.1016/j.soilbio.2021.108338

[41] Wen T, Xie P, Yang S, Niu G, Liu X, Ding Z, Xue C, Liu YX, Shen Q, Yuan J (2022) ggClusterNet: an R package for microbiome network analysis and modularity-based multiple network layouts. iMeta 1:e32 38868720 10.1002/imt2.32 PMC10989811

[42] Deng Y, Jiang Y-H, Yang Y, He Z, Luo F, Zhou J (2012) Molecular ecological network analyses. BMC Bioinformatics 13:1–20 22646978 10.1186/1471-2105-13-113 PMC3428680

[43] Sexton JP, Montiel J, Shay JE, Stephens MR, Slatyer RA (2017) Evolution of ecological niche breadth. Annu Rev Ecol Evol Syst 48:183–206

[44] Burns JH, Strauss SY (2011) More closely related species are more ecologically similar in an experimental test. Proc Natl Acad Sci 108:5302–5307 21402914 10.1073/pnas.1013003108 PMC3069184

[45] Larkin AA, Martiny AC (2017) Microdiversity shapes the traits, niche space, and biogeography of microbial taxa. Environmental Microbiology Reports 9:55–70 28185400 10.1111/1758-2229.12523

[46] Yang Y (2021) Emerging patterns of microbial functional traits. Trends Microbiol 29:874–882 34030967 10.1016/j.tim.2021.04.004

[47] Tian P, Razavi BS, Zhang X, Wang Q, Blagodatskaya E (2020) Microbial growth and enzyme kinetics in rhizosphere hotspots are modulated by soil organics and nutrient availability. Soil Biol Biochem 141:107662

[48] Xu J, Zhang Y, Zhang P, Trivedi P, Riera N, Wang Y, Liu X, Fan G, Tang J, Coletta-Filho HD (2018) The structure and function of the global citrus rhizosphere microbiome. Nat Commun 9:4894 30459421 10.1038/s41467-018-07343-2 PMC6244077

[49] Ling N, Wang T, Kuzyakov Y (2022) Rhizosphere bacteriome structure and functions. Nat Commun 13:836 35149704 10.1038/s41467-022-28448-9 PMC8837802

[50] Li E, de Jonge R, Liu C, Jiang H, Friman V-P, Pieterse CMJ, Bakker PAHM, Jousset A (2021) Rapid evolution of bacterial mutualism in the plant rhizosphere. Nat Commun 12:3829. 10.1038/s41467-021-24005-y 34158504 10.1038/s41467-021-24005-y PMC8219802

[51] Ellermann M, Sperandio V (2020) Bacterial signaling as an antimicrobial target. Curr Opin Microbiol 57:78–86 32916624 10.1016/j.mib.2020.08.001 PMC8163024

[52] Chen Y-J, Leung PM, Cook PL, Wong WW, Hutchinson T, Eate V, Kessler AJ, Greening C (2022) Hydrodynamic disturbance controls microbial community assembly and biogeochemical processes in coastal sediments. ISME J 16:750–763 34584214 10.1038/s41396-021-01111-9 PMC8857189

[53] Li J, Mara P, Schubotz F, Sylvan JB, Burgaud G, Klein F, Beaudoin D, Wee SY, Dick HJ, Lott S (2020) Recycling and metabolic flexibility dictate life in the lower oceanic crust. Nature 579:250–255 32161389 10.1038/s41586-020-2075-5

[54] Schäfer M, Pacheco AR, Künzler R, Bortfeld-Miller M, Field CM, Vayena E, Hatzimanikatis V, Vorholt JA (2023) Metabolic interaction models recapitulate leaf microbiota ecology. Science 381:eadf5121. 10.1126/science.adf5121 37410834 10.1126/science.adf5121

[55] Hammer BK, Bassler BL (2003) Quorum sensing controls biofilm formation in Vibrio cholerae. Mol Microbiol 50:101–104 14507367 10.1046/j.1365-2958.2003.03688.x

[56] Neelapu NRR, Dutta T, Challa S (2018) Quorum sensing and its role in agrobacterium mediated gene transfer. In: Pallaval Veera B (ed) Implication of Quorum Sensing System in Biofilm Formation and Virulence. Springer Singapore, Singapore, pp 259–275

[57] Nazir R, Warmink JA, Boersma H, Van Elsas JD (2009) Mechanisms that promote bacterial fitness in fungal-affected soil microhabitats. FEMS Microbiol Ecol 71:169–185 20002182 10.1111/j.1574-6941.2009.00807.x

[58] Fierer N, Bradford MA, Jackson RB (2007) Toward an ecological classification of soil bacteria. Ecology 88:1354–1364. 10.1890/05-1839 17601128 10.1890/05-1839

[59] Qiu L, Zhang Q, Zhu H, Reich PB, Banerjee S, van der Heijden MGA, Sadowsky MJ, Ishii S, Jia X, Shao M, Liu B, Jiao H, Li H, Wei X (2021) Erosion reduces soil microbial diversity, network complexity and multifunctionality. ISME J 15:2474–2489. 10.1038/s41396-021-00913-1 33712698 10.1038/s41396-021-00913-1 PMC8319411

[60] Simon A, Bindschedler S, Job D, Wick LY, Filippidou S, Kooli WM, Verrecchia EP, Junier P (2015) Exploiting the fungal highway: development of a novel tool for the in situ isolation of bacteria migrating along fungal mycelium. FEMS Microbiol Ecol 91. 10.1093/femsec/fiv116 10.1093/femsec/fiv116 26432804

