# Supplementary material for: Deciphering Microbial Adaptation in the Rhizosphere: Insights into Niche Preference, Functional Profiles, and Cross-Kingdom Co-occurrences
Source: Microb Ecol. 2024 May 21;87(1):74. doi: 10.1007/s00248-024-02390-3 (PMC11108897; doi:10.1007/s00248-024-02390-3)
Supplement: Supplementary file 2 — Supplementary file2 (DOCX 895 KB) [file 248_2024_2390_MOESM2_ESM.docx]

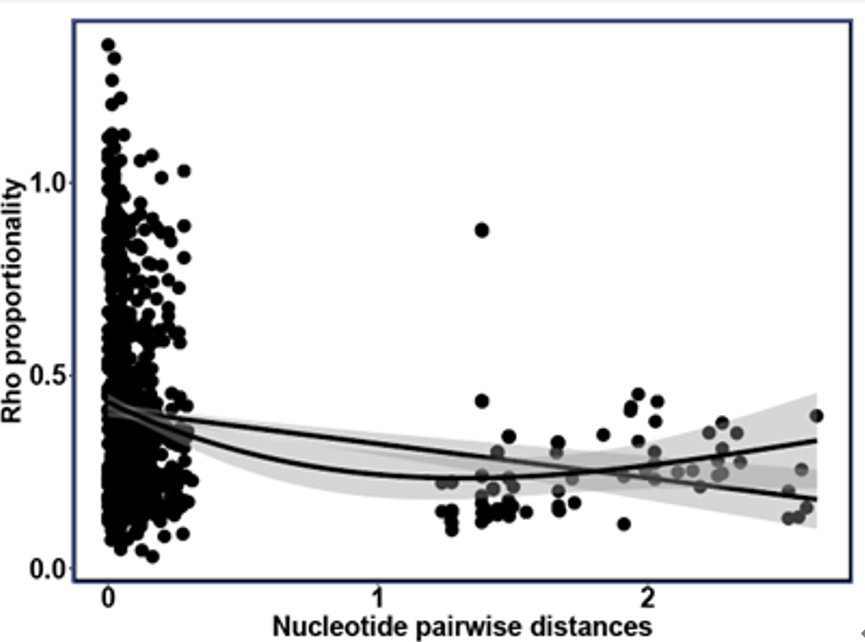


**Fig.S1 The relationship of Rho values and nucleotide distances**


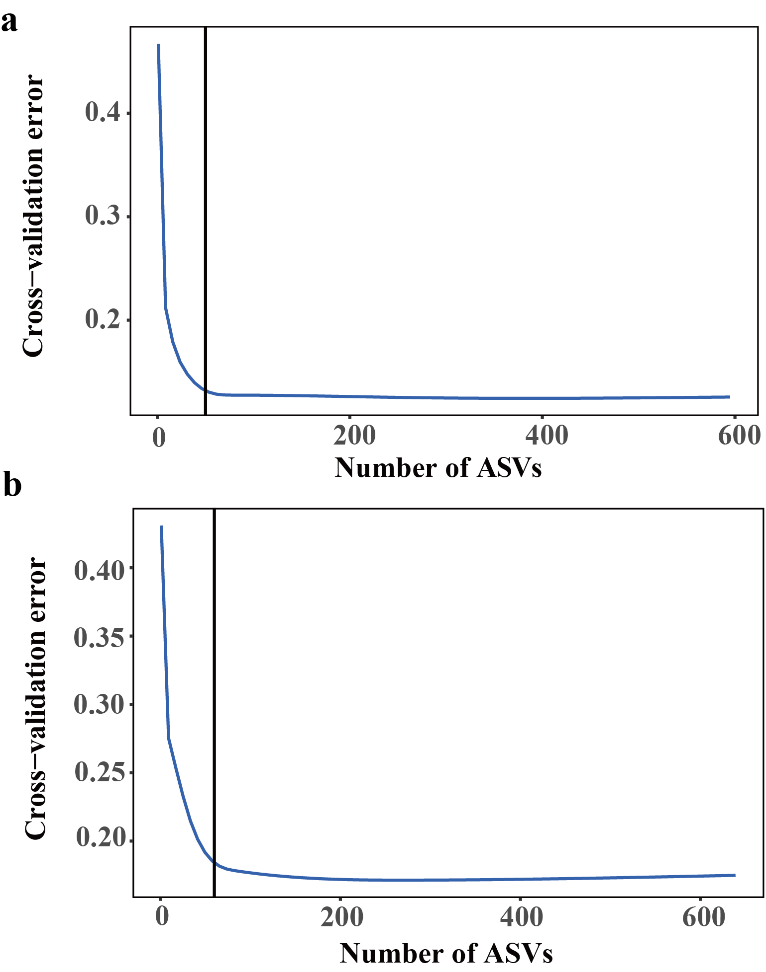


**Fig. S2 Cross-validation curve of bacteria (a) and fungi (b) shows the relationship between the model error and the number of ASVs used for fitting**


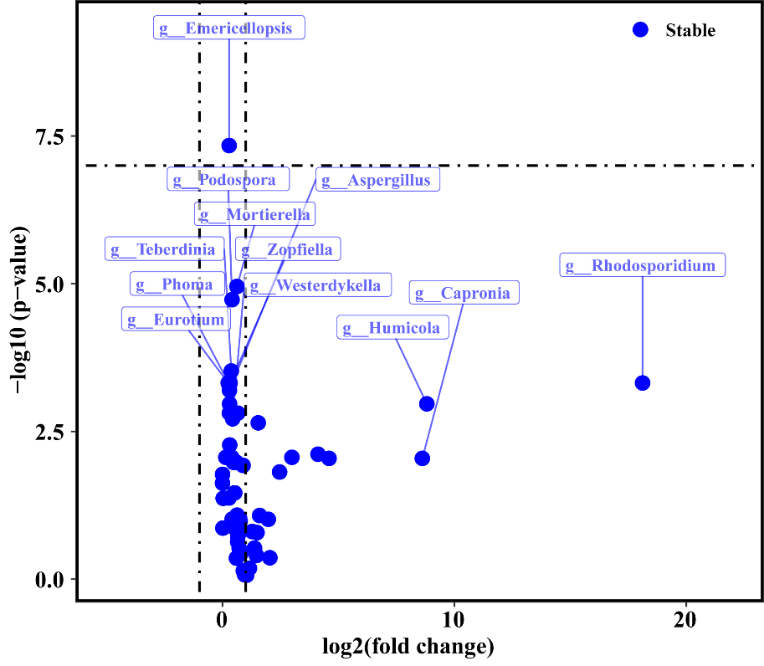


**Fig. S3 Differential genera of fungi between bulk and rhizosphere soil samples. No significant enrichment of fungal genera in the rhizosphere.**


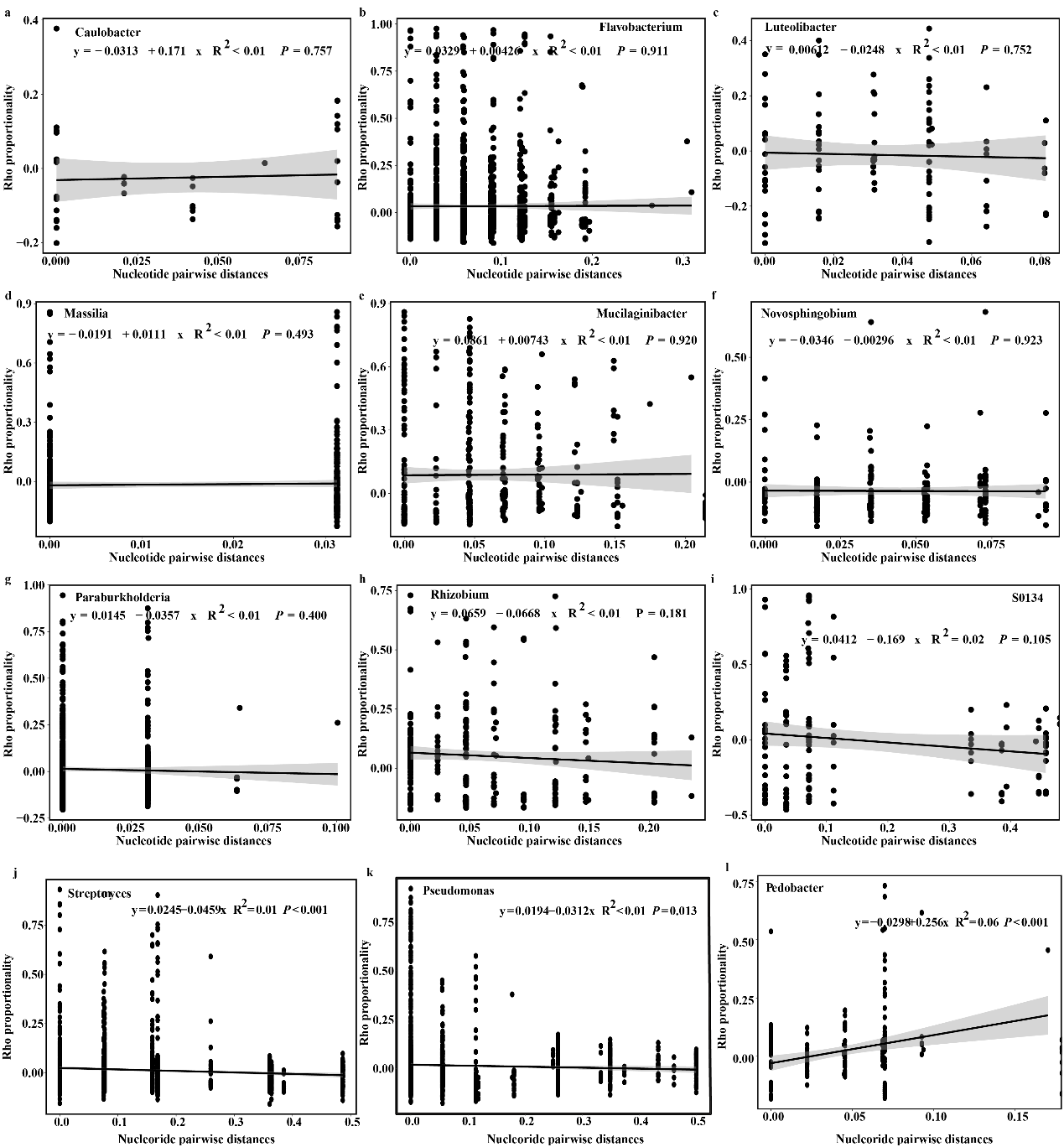


**Fig.S4 Relationship between the Rho proportionality and the nucleotide divergence. The closely related ASV is defined as nucleotide distance less than 0.5. Gray and black lines represent the linear relationship between the two variables. The *P* value is displayed for the regressions.**


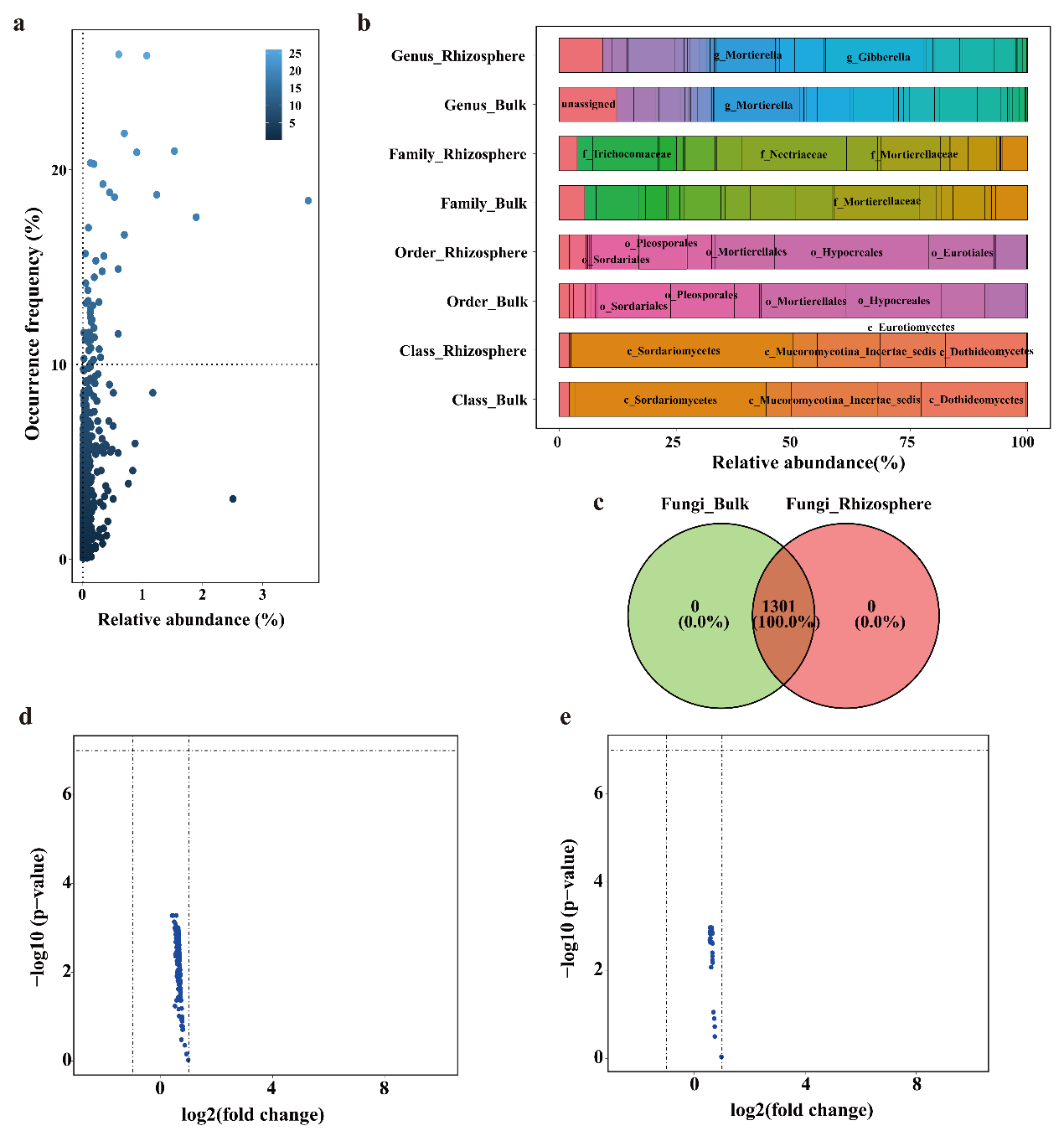


**Fig. S5 Function profiles of fungal abundant species. The fungal abundant ASVs is defined as the relative abundance and occurrence frequency (a) and Venn diagram shows the shared ASVs in bulk and rhizosphere samples (c). Bar chart of relative abundance at different taxonomic level of fungal abundant species (b). There was no statistically significant difference of KOs and pathway of core fungal ASVs between bulk and rhizosphere soils (d, e).**


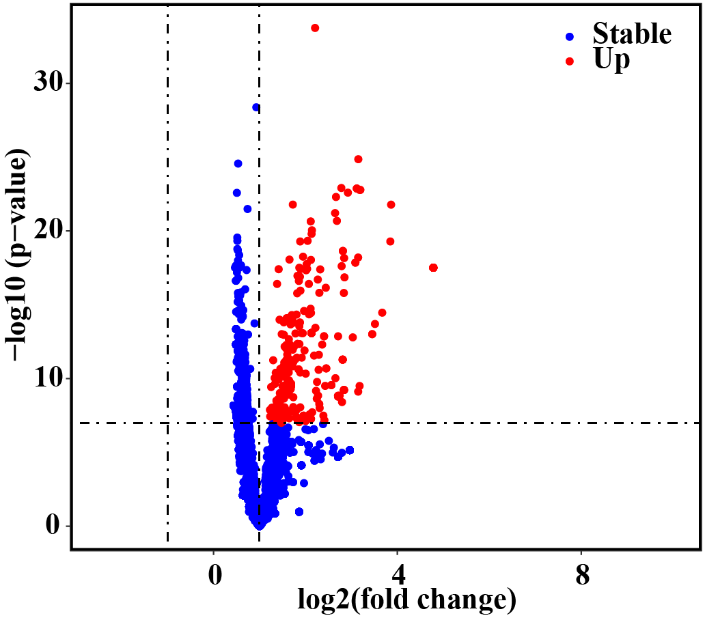


**Fig. S6 Differential KOs between bulk and rhizosphere samples.** **Differential KOs of bacteria are marked in red font in volcano plots.**


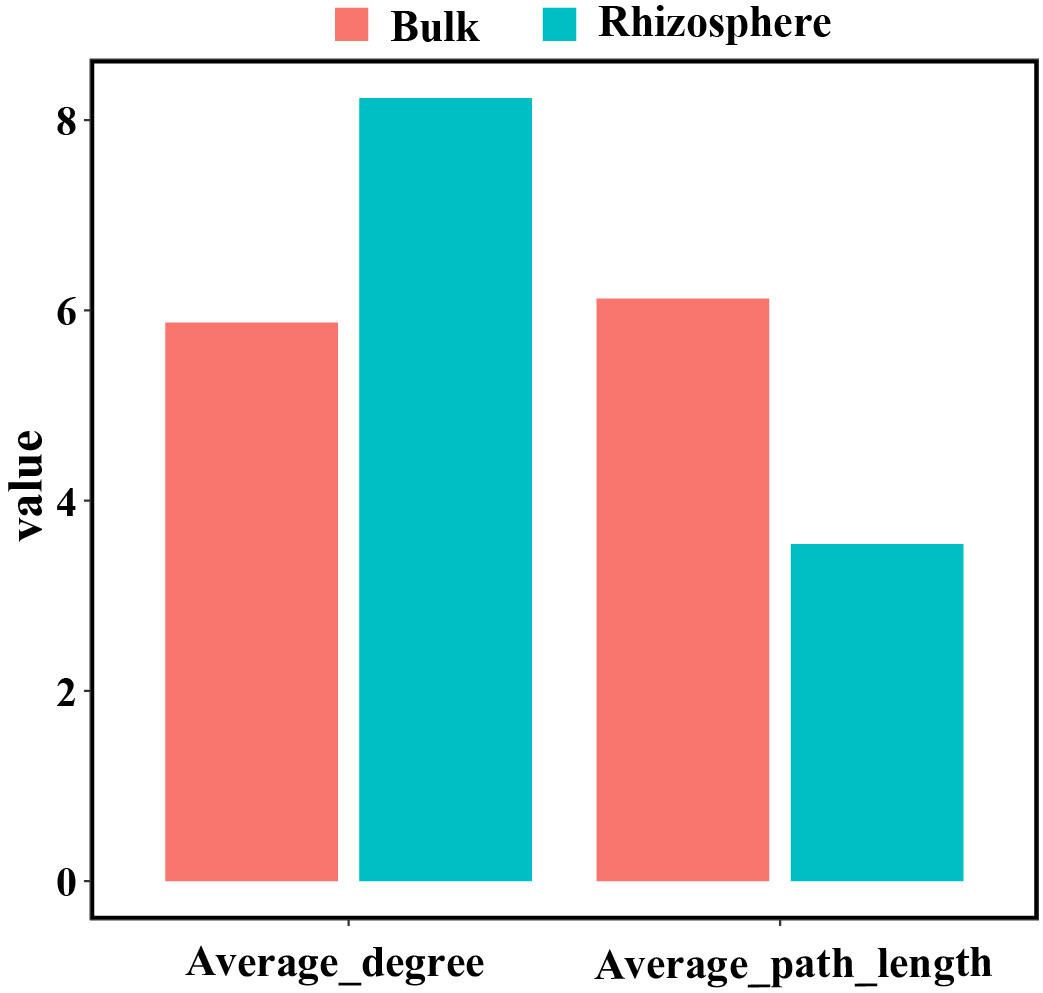


**Fig. S7 Network topologies (average degree and average path length) of bulk and rhizosphere cross-kingdom networks**
